# Enhanced discrimination of *Clostridioides difficile* transmission using whole-genome sequencing and *in silico* multi-locus variable number tandem repeat analysis

**DOI:** 10.1128/spectrum.03287-24

**Published:** 2025-10-27

**Authors:** Alexander J. Sundermann, Emma G. Mills, Vatsala Rangachar Srinivasa, Marissa P. Griffith, Deena Ereifej, Kady D. Waggle, Nathan J. Raabe, Graham M. Snyder, Daria Van Tyne, Lora Lee Pless, Lee H. Harrison

**Affiliations:** 1 Microbial Genomic Epidemiology Laboratory, Center for Genomic Epidemiology, University of Pittsburgh 6614, Pittsburgh, Pennsylvania, USA; 2 Division of Infectious Diseases, University of Pittsburgh School of Medicine 12317 , Pittsburgh, Pennsylvania, USA; 3 Department of Epidemiology, School of Public Health, University of Pittsburgh 6614, Pittsburgh, Pennsylvania, USA; 4 Department of Infection Control and Hospital Epidemiology, UPMC Presbyterian Hospital, Pittsburgh, Pennsylvania, USA; The George Washington University School of Medicine and Health Sciences, Washington, DC, USA

**Keywords:** *Clostridioides difficile*, multi-locus variable number tandem repeat analysis, whole-genome sequencing, genomic epidemiology, hospital-associated infections, outbreaks

## Abstract

Whole-genome sequencing (WGS) is used to establish genetic relatedness of bacteria and track outbreaks in healthcare settings. While WGS provides sufficient discriminatory power to make inferences about genetic relatedness and transmission for most bacterial species, WGS for *Clostridioides difficile* often fails to do so, even at low single-nucleotide polymorphism (SNP) differences. Multi-locus variable number tandem repeat analysis (MLVA), which analyzes rapidly mutating tandem repeat loci, has previously been shown to be useful for this purpose for *C. difficile*. We investigated whether *in silico* MLVA can further elucidate genetic relatedness of *C. difficile* clusters identified by short-read WGS but lacking epidemiological links. Potential healthcare-associated toxin-positive *C. difficile* isolates were collected at our hospital from November 2016 to November 2019. Short-read WGS was performed on the Illumina platform to cluster isolates with ≤2 SNPs, and Nanopore long-read sequencing was used to resolve MLVA loci within these clustered isolates. Among 666 isolates, 62 unique patient isolates met the ≤2 SNP criterion and underwent MinION sequencing. Of the 105 pairs with 0–2 SNP differences, 79.0% had a summed tandem-repeat difference (STRD) of 0–5, 10.5% had an STRD of 6–10, and 10.5% had an STRD of 11–20. A significant correlation was found between a lower STRD value and the presence of a unit/procedure-based epidemiological link within low SNP clusters (odds ratio: 0.45; 95% CI: 0.29–0.70). Our findings demonstrate that MLVA provides additional genomic discrimination for closely related *C. difficile* isolates identified by WGS, enhancing outbreak investigation precision.

**IMPORTANCE:**

*Clostridioides difficile* is a leading cause of healthcare-associated infections, often spreading undetected within hospitals. To track its transmission, hospitals increasingly rely on bacterial genetic sequencing, but this approach is often not discriminatory enough for identifying the spread of this organism between patients. In this study, we applied an additional genetic method that looks at highly changeable regions of the bacteria’s DNA to improve the detection of likely transmission events. By combining two sequencing techniques, we were able to separate seemingly related infections that were not actually linked in the hospital. This enhanced resolution can help infection prevention teams focus their investigations and stop real outbreaks more efficiently, improving patient safety. Our findings support the use of this combined sequencing strategy in routine hospital surveillance and show how it can fill important gaps when standard methods are not sufficient.

## INTRODUCTION

Whole-genome sequencing (WGS) has become the primary approach for establishing genetic relatedness of bacteria. This method enables investigators to detect and track outbreaks of pathogens throughout healthcare settings and the community. Our hospital uses WGS surveillance in combination with machine learning of the electronic health record to identify otherwise undetected outbreaks and determine the most likely transmission route, respectively (1). Although we do not use a strict threshold of single-nucleotide polymorphism (SNP) differences to infer transmission, we have generally found that ≤10–15 SNPs is a valid initial threshold for determining genetic relatedness with supportive epidemiological evidence (1–4). However, the *Clostridioides difficile* genome is relatively slow to mutate and in spore form does not replicate, which results in lower thresholds of SNP distance to establish genetic relatedness (5). Accordingly, it is common to not find an epidemiologic link for isolates that appear to be related genetically, even at low SNP thresholds (e.g., ≤2 SNPs), indicating that SNP-based analyses provide insufficient discriminatory power for this organism (5, 6).

Tandem repeat loci have been referred to as “mutational hotspots” because they are among the most rapidly changing regions of the bacterial genome (7). These loci have a propensity for strand-slippage replication as well as recombination mechanisms that lead to a decrease or increase in the number of repeats. Tandem repeats allow bacteria to rapidly adapt to their environment by regulating gene expression through phase variation and are also a useful tool for epidemiologic tracking. Multi-locus variable number tandem repeat analysis (MLVA) was first developed for *Bacillus anthracis*, an organism for which epidemiologically unrelated isolates could not be distinguished based upon molecular epidemiologic methods that were available at the time (8). Traditional MLVA was performed by PCR amplification of tandem repeat loci and inferring the number of repeats at each locus based on the fragment size of the PCR product. The summed tandem repeat difference (STRD) between two isolates is calculated by adding the number of repeat differences at each of the MLVA loci and can be used to make inferences about transmission (9). MLVA was replaced by WGS as the cost and availability of WGS have made the technology more accessible. However, using WGS, MLVA theoretically could be performed *in silico* from WGS data (10).

An MLVA assay for *C. difficile* was previously developed and used by our group and others to assess the relatedness and hospital transmission of *C. difficile* isolates before WGS became widely available (9, 11, 12). The assay was performed using PCR amplification followed by fragment size analysis. We developed our initial assay using Tandem Repeats Finder software and included repeat elements in the *C. difficile* 630 chromosome that (i) had more than one allele among the isolates studied, (ii) were sufficiently stable and discriminatory, and (iii) were epidemiologically useful for identifying transmission in the hospital (9, 13). MLVA was initially considered a temporary bridging technology between band-based molecular typing methods and WGS, with the expectation that it would become obsolete once WGS became widely available.

However, with the widespread availability of WGS, an *in silico* approach can be used to elucidate MLVA loci. A prior study determined that WGS and MLVA were generally concordant for determining *C. difficile* genetic relatedness (10). However, the authors sometimes found discordance between the two methods, with some isolates having ≤2 SNPs (suggesting transmission) but an STRD ≥10 (suggesting that transmission was unlikely). These results suggested that MLVA may increase discriminatory power among isolates that appear to be highly related by WGS.

In using routine WGS surveillance for identifying otherwise unrecognized outbreaks of *C. difficile* in our hospital, we have identified many genetically related clusters (≤2 SNPs) for which an epidemiologic link could not be identified (1). We therefore investigated whether (i) *in silico* MLVA could provide discriminatory power in addition to WGS, and (ii) whether this approach could be useful epidemiologically in the setting of genomic surveillance for *C. difficile* transmission.

## MATERIALS AND METHODS

### Study setting

This study was performed at UPMC Presbyterian Hospital, an adult tertiary care hospital with 699 total beds, 134 critical care beds, and over 400 annual solid organ transplants.

### Isolate collection

Culture-independent diagnostic test-positive *C. difficile* stool specimens were collected between November 2016 and November 2019, as previously described (1). Clinical criteria for testing required at least three unformed stools and a clinical indication of colitis. From 2016 through 2018, *C. difficile* testing was performed using PCR for detection of gene toxin; in 2019, an enzyme immunoassay to detect glutamate dehydrogenase and *C. difficile* toxin was used, with PCR for detection of gene toxin to resolve discordant results (glutamate dehydrogenase positive and toxin negative). We then performed a culture of these specimens for *C. difficile*. Inclusion criteria were hospital admission or observation ≥3 days before the collection date and/or a recent inpatient or outpatient encounter in 30 days before the collection date. A total of 666 isolates were collected and analyzed.

### Selection and characteristics of MLVA loci used in this study

We used seven MLVA loci that we previously identified and validated as being useful for establishing genetic relatedness of *C. difficile* for studies of transmission (9, 11, 12). The characteristics of the loci are shown in Table 1.

**TABLE 1:** Characteristics of the multi-locus variable tandem repeat analysis (MLVA) loci used in this study. Adapted from reference 9

			Prior study (9)		Current study		
MLVA locus	Genome location^*a*^	Size (bp)	Copy no. range^*b*^	No. of alleles^*b*^	Copy no. range^*c*^	No. of alleles^*c*^	Tandem repeat sequence
CDR4	755,721	6	3–41	28	15–37	19	TTGCTC
CDR5	692,929	8	0–17	9	3–11	5	TATATTG/AG
CDR9	664,660	8	3–34	17	3–20	15	TAAAAGAG
CDR48	167,124	7	3–13	9	5–11	6	ATAGATT
CDR49	3,688,632	7	1–31	21	8–22	12	AT/AC/TTTCT
CDR59	771,338	11	5–10	6	4–12	5	TAAG/ATATA/GGAT/C
CDR60	677,132	17	2–27	18	2-21	9	G/ATAAA/GTAGGATG/ATAAAA

^*a*^ Genome location based on *C. difficile* 630 sequence (https://www.sanger.ac.uk/).

^*b*^ Based on analysis of 87 isolates in our prior study (9).

^*c*^ Based on analysis of 62 isolates in the current study.

### WGS and assembly methods

#### Short-read WGS

DNA was prepared for Illumina sequencing using the Illumina DNA Prep Kit, and libraries were sequenced on a NextSeq 550 (2 × 150 bp; v2.5 300-cycle kit; Illumina, San Diego, CA). Samples were demultiplexed using bcl2fastq (v2.20, Illumina), followed by genome assembly using Unicycler v0.5.1 (14). Annotation was performed with Prokka v1.14 (15), and multi-locus sequence types (STs) were assigned using the PubMLST typing scheme (https://github.com/tseemann/mlst) (16). Data quality control included removal of genomes with >350 contigs, total genome length <3.2 Mbp or >4.8 Mbp, or <35× average sequencing depth.

Pairwise core genome SNPs (cgSNPs) were determined using two approaches: (i) Snippy-core was run for each ST using a reference genome selected based on the highest *N*_50_ within each ST (v4.3.0; https://github.com/tseemann/snippy) and (ii) a reference-free split k-mer approach was used with SKA v1.0 (17). Genetically related clusters were identified using hierarchical clustering with average linkage, prioritizing the minimum SNP value from either Snippy or SKA for each pair. Clusters were defined as isolates from >1 patient having ≤2 pairwise cgSNPs (18).

#### Long-read WGS

Long-read sequencing was performed for the 62 samples that formed transmission clusters based on short-read sequencing data. Nanopore DNA sequencing libraries were prepared using the SQK-RBK004 multiplex rapid gDNA barcoding kit and were sequenced on an Oxford Nanopore Technologies (ONT) MinION Mk1C device using R9.4.1 flow cells. Base calling and demultiplexing were conducted using Albacore v2.3.3 or Guppy v2.3.1. All 62 samples met quality control criteria (≥10× average read depth) for further analyses.

#### Hybrid and polished long-read assembly methods

Hybrid assemblies were created using Unicycler 0.5.1 by integrating both ONT and Illumina reads for each of the 62 clustered isolates (14). Additionally, long-read-first assemblies were generated using ONT long-reads with Flye, followed by polishing with Illumina short-reads using Polypolish v0.5.0 (19, 20).

#### Analysis of optimal method for MLVA locus resolution

To determine the optimal method for resolving MLVA loci, we compared three sequencing approaches using Sanger sequencing as the gold standard: (i) short-read sequencing alone (Illumina, assembled with Unicycler), (ii) hybrid assembly (Illumina + ONT, assembled with Unicycler), and (iii) long-read-first assemblies polished with short reads (ONT + Illumina, assembled with Flye and polished with Polypolish) (20). Sanger sequencing was performed using dye-labeled dideoxynucleotide chain termination with forward and reverse primers in separate reactions (21, 22). We evaluated three loci (CD4R4, CDR9, and CDR60) in five isolates.

#### STRD calculation and epidemiologic categorization

STRD was calculated pairwise for all isolates in a cluster by summing tandem repeat count differences across all seven MLVA loci. For example, if Isolate 1 had repeat counts of 10, 5, 8, 6, 7, 12, 9 and Isolate 2 had 12, 5, 9, 6, 10, 15, and 11, the STRD would be: |10 − 12| + |5 − 5| + |8 − 9| + |6 − 6| + |7 − 10| + |12 − 15| + |9 − 11| = 11. STRDs were categorized based on prior validation studies as follows: Indistinguishable (STRD = 0), Highly related (STRD = 1–2), Closely related (STRD = 3–4), Possibly related (STRD = 5–10), and Unrelated (STRD >10) (9).

#### Transmission analysis

Epidemiological links were determined using methods as previously described (1). Briefly, clusters were reviewed for unit commonalities, shared procedures, and common healthcare workers prior to their positive test dates. Additionally, a machine learning algorithm utilizing electronic health record charge codes and healthcare worker data were applied as an adjunct to find other plausible routes of transmission (1). These routes were reviewed by infection prevention experts to: (i) identify the most likely transmission route among the possibilities suggested by the machine learning algorithm, and (ii) potentially find additional routes of transmission otherwise missed. Transmission routes were stratified into unit epidemiological commonalities alone and any (unit, procedural, or healthcare worker) epidemiological commonality.

#### Statistical analyses

Regression analysis was performed for the correlation of STRD (as a continuous variable) to determine the presence of any epidemiological link or a unit-based epidemiological link within *C. difficile* clusters (binary outcomes). The outcome model’s performance was assessed using the area under the curve (AUC) of the receiver operating characteristic (ROC) curve. Additionally, an ANOVA with Tukey’s HSD test was conducted to measure the association between STRD and pairwise SNP distance to determine if there were significant differences in STRD across the different levels of pairwise SNP distance. All analysis was performed using SAS v.9.4.

## RESULTS

**Fig 1:**
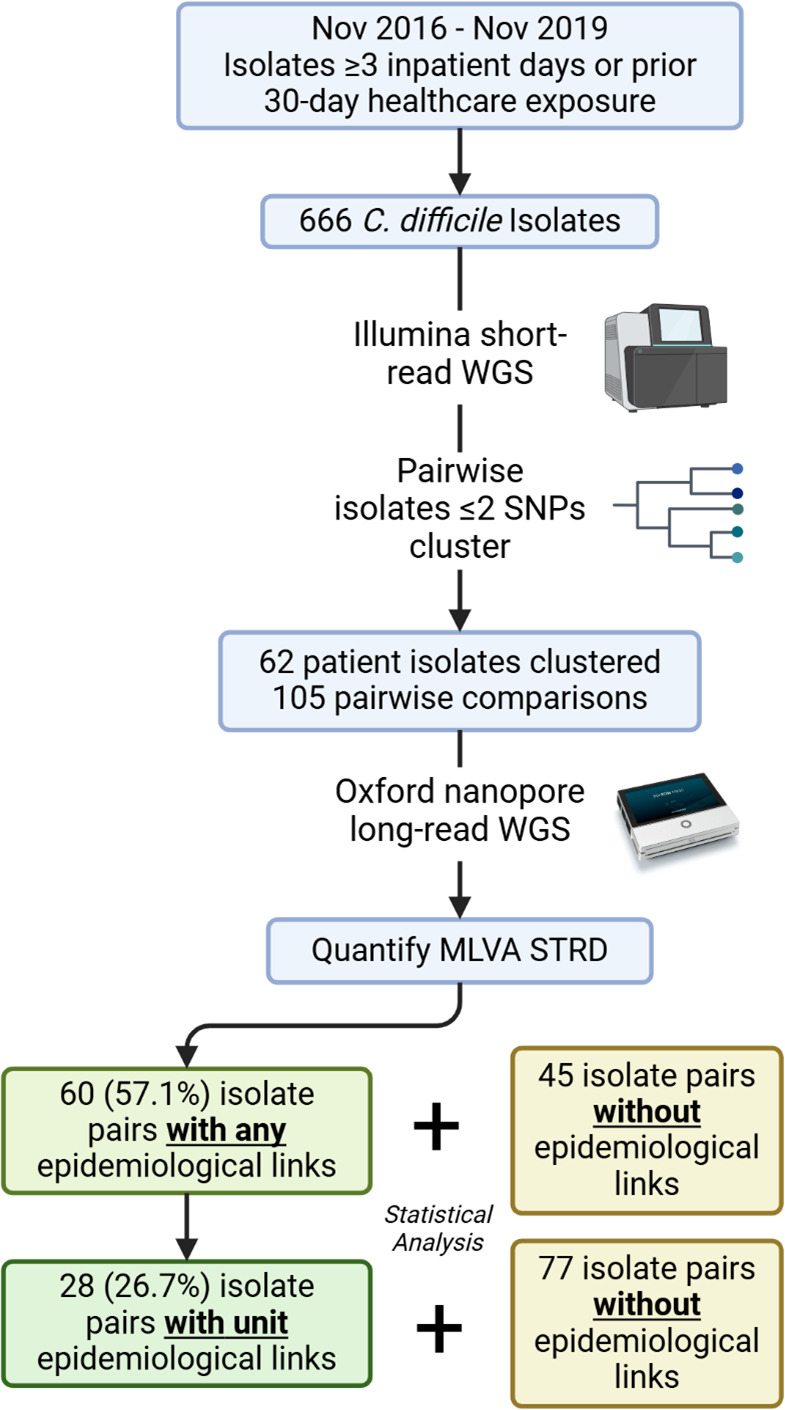
Process and pipeline for multi-locus variable number tandem repeat analysis (MLVA) summed tandem repeat difference (STRD) analysis.

Short-read WGS on the Illumina platform was performed on 666 isolates during the 36-month study period (Fig. 1). Pairwise SNP analysis identified 62 isolates among 20 clusters (range: 2–10 patients) that were closely related (≤2 SNPs), and these isolates were sequenced on the ONT platform; the mean contig count was 144.3 (SD = 69.8) and 8.4 (SD = 29.1) for short- and long-read assemblies, respectively. Comparison with Sanger sequencing showed that ONT long-read-first assemblies polished with Illumina short reads provided the most accurate MLVA locus resolution. The results were concordant, with the exception of a difference of a single tandem repeat for isolate CD07919 at the CD4 locus (Table S2); this may represent *in vitro* evolution. In contrast, Illumina short-read results differed substantially from Sanger sequencing the majority of the time. Determining MLVA loci from hybrid assemblies performed similarly to the Illumina short-read assembly results (data not shown). Based on these results, all subsequent analyses of MLVA loci were based on polished long-read-first assembly results.

Using the polished ONT assemblies, among the 105 isolate pairs with 0–2 SNP differences, 83 pairs (79.0%) had an STRD of 0–5, 11 pairs (10.5%) had an STRD of 6–10, and 11 pairs (10.5%) had an STRD of 11–20. The number of tandem repeats at each locus is provided in Table S3.

**Fig 2:**
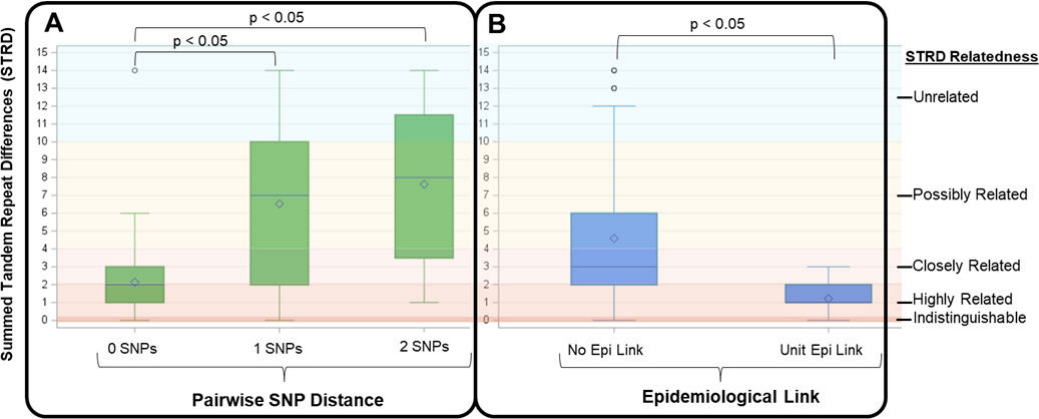
(**A**) Summed tandem repeat difference (STRD) for each pairwise SNP distance (0, *n* = 72 pairs; 1, *n* = 17 pairs; and 2, *n* = 16 pairs). (**B**) Relationship between STRD for presence of a unit/procedure epidemiological link (*n* = 28 pairs) versus no epidemiologic link (*n* = 77 pairs), among within ≤2 SNP *C. difficile* clusters. Diamonds and horizontal lines represent the mean and median values, respectively.

We stratified isolate pairs first by whether they had any epidemiological link and then further into those that had a unit-based epidemiological link (defined as patients who had a shared unit stay in common within 100 days). Overall, 60 (57.1%) isolate pairs had an identified epidemiological link, while 28 (26.7%) had a unit-based epidemiological link. Logistic regression analysis found no significant association between MLVA STRD and the presence of any epidemiological link within these clusters (odds ratio [OR]: 0.94; 95% confidence interval [CI]: 0.85–1.04). However, regression analysis revealed a significant association between a lower MLVA value and the presence of a unit-based epidemiological link within low SNP clusters (OR: 0.45; 95% CI: 0.29–0.70) (Fig. 2), that is, for each incremental increase of 1 in STRD, the odds of having a unit-based epidemiological link decrease by 55%. The AUC for the ROC curve was 0.80, indicating good discriminatory ability of the model. ANOVA analysis revealed significant differences in STRD when comparing pairwise SNP distances. Specifically, there was a significant difference in STRD between 0 and 1 SNP pairwise comparisons (*P* < 0.05; 95% CI: 2.6–6.2) and between 0 and 2 SNP pairwise comparisons (*P* < 0.05; 95% CI: 3.5–7.5). However, there was no significant difference in STRD between 1 and 2 SNP pairwise comparisons (*P* > 0.05; 95% CI: −1.4 to 3.6).

## DISCUSSION

In this study, we demonstrate that MLVA provides additional genomic discrimination in *C. difficile* isolates that are closely related via WGS. Using this method, many *C. difficile* isolates in SNP-defined clusters without any epidemiological links were removed from clusters when an additional lower STRD threshold was applied. This application may enable hospital epidemiologists to better prioritize outbreak investigations that have lower STRDs, thus directing investigation resources more effectively towards suspected outbreaks where transmission may truly be occurring. Our data suggest that an STRD of approximately 3 would be a reasonable cutoff, although prospective validation of this approach is required.

Previously, the generation of *in silico* MLVA data were not feasible for routine practice due to the need for multiple pipelines and the associated duplicative costs. Our analysis shows that MLVA cannot be accurately determined via short-read sequencing alone; instead, a polished long-read-first sequencing approach is required. Institutions currently performing WGS surveillance should consider incorporating this method into their *C. difficile* investigations. Going forward, our WGS surveillance program has adopted the approach of performing long-read WGS on short-read WGS clusters to focus the investigation on isolate pairs with small STRDs.

There are limitations to our study. First, we did not sample asymptomatic carriers who could act as transmission sources. Second, although we conducted an exhaustive investigation into transmission routes, there may have been transmissions that were unidentified through our electronic health record review. Third, we acknowledge that our analysis uses STRD as a cumulative measure of repeat differences, without accounting for the possibility that larger differences at a single locus may represent a single evolutionary event. While this simplification may overestimate genetic distance, our prior work involving environmental and serial patient isolates suggests that observed mutational differences are typically limited to single repeat changes (9). Nonetheless, here we demonstrate that MLVA has the ability to separate closely related *C. difficile* isolates defined by WGS without epidemiological links. Implementing *in silico* MLVA into our outbreak detection pipeline can better direct infection prevention resources and guide interventions to halt transmission, thereby improving patient safety.

## Data Availability

Illumina and ONT sequence data are available in the National Center for Biotechnology Information (NCBI) BioProject PRJNA475751 (see Table S1 for accession identifiers) (18). Scripts for MLVA locus identification and STRD calculations are available at https://github.com/mpgriffith/mlva.

## SUPPLEMENTAL MATERIAL

Table S1 spectrum.03287-24-s0001.xlsx National Center for Biotechnology Information Sequence Read Archive accession numbers for study isolates.

Table S2 spectrum.03287-24-s0002.xlsx Comparison of differences in the number of tandem repeats at three MLVA loci for five isolates, determined by short-read (Illumina), long-read (ONT), and Sanger sequencing.

Table S3 spectrum.03287-24-s0003.xlsx Number of of tandem repeats at each MLVA locus.

ASM does not own the copyrights to Supplemental Material that may be linked to, or accessed through, an article. The authors have granted ASM a non-exclusive, world-wide license to publish the Supplemental Material files. Please contact the corresponding author directly for reuse.
